# Psychotic disorders hospitalizations associated with cannabis abuse or dependence: A nationwide big data analysis

**DOI:** 10.1002/mpr.1813

**Published:** 2019-12-05

**Authors:** Manuel Gonçalves‐Pinho, Miguel Bragança, Alberto Freitas

**Affiliations:** ^1^ Department of Community Medicine, Information and Health Decision Sciences (MEDCIDS), Faculty of Medicine University of Porto Porto Portugal; ^2^ 2D4H – Secondary Data for Healthcare Research Center for Health Technology and Services Research (CINTESIS) Porto Portugal; ^3^ Department of Clinical Neurosciences and Mental Health, Faculty of Medicine University of Porto Porto Portugal

**Keywords:** cannabis, epidemiology, health service, psychoses, public mental health

## Abstract

**Objectives:**

We aimed to describe and correlate the hospital panorama of psychotic disorders (PD) with cannabis use (CU) trends in all Portuguese public hospitals.

**Methods:**

We conducted a retrospective observational study that analysed all hospitalizations that occurred in Portuguese public hospitals from 2000 to 2015. Hospitalizations with a primary diagnosis of PD or schizophrenia were selected based on Clinical Classification Software diagnostic single‐level 659. Episodes associated with CU were identified by the International Classification of Diseases Version 9, Clinical Modification code 304.3/305.2 that correspond to cannabis dependence/cannabis abuse.

**Results:**

The number of hospitalizations with a primary diagnosis of PD and schizophrenia associated with CU rose 29.4 times during the study period, from 20 to 588 hospitalizations yearly (2000 and 2015, respectively) with a total of 3,233 hospitalizations and an average episode cost of €3,500. Male patients represented 89.8% of all episodes, and the mean/median age at discharge were 30.66/29.00 years, respectively. From all hospitalizations with a primary diagnosis of PD or schizophrenia, the ones with a secondary diagnosis of CU rose from 0.87% in 2000 to 10.60% in 2015.

**Conclusions:**

The increase on secondary diagnosis coding and the change on cannabis patterns of consumption in Portuguese population with an increasing frequency of moderate/high dosage cannabis consumers may explain the rise on PD hospitalizations.

## 1 INTRODUCTION

Cannabis is one of the most commonly used recreational drugs and is also utilized for its medical properties (Carliner, Brown, Sarvet, & Hasin, 2017; Davis, 2016). The primary psychoactive compound of cannabis is delta 9‐tetrahydrocannabinol that activates cannabinoid receptors. These receptors when activated will interfere in brain activity, more specifically in areas of the brain that are responsible for cognition, perception, anxiety, fear, memory and reward (Grotenhermen, 2003).

Most studies analysing cannabis use (CU) impact on psychotic disorders (PD) identify two possibilities of relation: (a) first, CU as a contributing cause to initiate a PD and, (b) second, a shared vulnerability between CU and the beginning of a PD, where the risk factors for the development of a PD may also play a role in the presence of addictive behaviours (Ksir & Hart, 2016).

PD are one of the most serious group of medical conditions that may appear after CU and may generate comorbidities in an acute or chronic timeline (Gage, Hickman, & Zammit, 2016). Although it is difficult to establish a direct causal link between CU and PD, a large number of observational studies have found a clear association between CU and schizophrenia and the development of psychotic symptoms (Colizzi et al., 2018; Ferdinand et al., 2005; Hall & Degenhardt, 2000; Miettunen et al., 2008; Ortiz‐Medina et al., 2018; Regier et al., 1990; Semple, McIntosh, & Lawrie, 2005). Patients diagnosed with a first episode of psychosis or with schizophrenia are more likely to report current or prior use of cannabis, compared with the general population. Cannabis properties also play a role with the incidence of a first psychotic event being linked to the dosage of the cannabis used by the patient (Andreasson, Allebeck, Engstrom, & Rydberg, 1987; Di Forti et al., 2019; Roncero et al., 2018; Zammit, Allebeck, Andreasson, Lundberg, & Lewis, 2002). Continued CU not only might play a role on the first psychotic event but also affects negatively the prognosis of a patient after the first episode of psychosis, increasing the relapse rate (Schoeler et al., 2016). According to Tennant et al., PD related with CU may occur in three distinct situations: (a) auto‐limited psychosis caused by acute cannabis consumption that ends after stopping the consumption, (b) psychosis that develops during CU that requires medical treatment or hospitalization even after stopping the consumption, and (c) psychosis that manifests years after CU but is likely directly related to it (Rylander, Winston, Medlin, Hull, & Nussbaum, 2018; Tennant, 2005).

CU is linked to an increase in hospital visits and in the utilization rates of emergency and hospital services for psychosis (Rylander et al., 2018). In France, emergency department visits associated with CU have increased, and in the United States, there was a significant increase on CU‐related hospitalizations in the recent years (Charilaou et al., 2017; Noel, Maghoo, Franke, Viudes, & Minodier, 2019). In times where many countries are considering the legalization of cannabis for recreative consumption, research regarding cannabis role in PD arises as an important tool to understand its true impact in terms of public and mental health. In Portugal, the proprietorship and consumption of cannabis was decriminalized in 2001 together with all other drugs and is only considered a crime if a single individual possesses more than 10 standard doses. The legalization of the use of cannabis for recreative consumption is currently being debated by all political parties. The estimated prevalence of CU at any period of life in the Portuguese population is 9.7%, a lower value when compared with the average European prevalence. When analysing the data on European patterns of cannabis consumption, 7.4% of the population in Europe consumed cannabis in 2018 (European Monitoring Centre for Drugs and Drug Addiction, 2019). However, regarding the moderate/high consumption of cannabis, it is estimated that Portugal has a higher prevalence of consumers when compared with the average European prevalence (2.3–3.2% vs. 1.0%; Serviço de Intervenção nos Comportamentos Aditivos e nas Dependências (SICAD), 2017).

The main goal of this study was to analyse clinical, demographic, and administrative trends regarding PD hospitalizations associated with cannabis abuse and/or dependence in the recent years in Portugal. The secondary goal of this study was to use secondary data such as administrative databases in Psychiatry and Epidemiology observational research.

## 2 MATERIALS AND METHODS

We conducted a retrospective observational study using a database provided by *Administração Central dos Serviços de Saúde* that contained all hospitalizations registered in Portuguese public hospitals from 2000 to 2015. We selected all hospitalizations associated with CU, identified based on International Classification of Diseases Version 9, Clinical Modification codes of diagnosis 304.3 and 305.2 that correspond to cannabis dependence and cannabis abuse, respectively. These codes belong to the larger group of neurotic disorders, personality disorders, and other nonpsychotic mental disorders in International Classification of Diseases Version 9, Clinical Modification. Hospitalizations with a primary diagnosis of schizophrenia and other PD defined by the Clinical Classification Software diagnostic single‐level 659 were selected, representing a broad group of diagnosis that gather all psychotic conditions (Appendix A).

Information regarding birth date, sex, primary and secondary diagnoses, admission date, discharge date, length of stay (LoS), discharge status, and hospital charges from each single hospitalization episode were gathered. Hospital charges were calculated from expenditure tables for the Portuguese National Health Service hospital reimbursements, as defined by governmental decree in 2009 (in Diário da República) and were estimated by using a diagnosis‐related groups‐based budget allocation model.

Descriptive statistical analyses, independent sample *t* tests, and linear regression models with 95% confidence intervals (CI) were performed to assess temporal trends in the number of admissions, age at admission, and LoS between 2000 and 2015, using IBM SPSS Statistics v.24 for Windows (Armonk, NY: IBM Corp).

## 3 RESULTS

A total of 3,233 hospitalizations with a primary diagnosis of PD or schizophrenia and with a secondary diagnosis of cannabis abuse or dependence occurred in all Portuguese public hospitals between the year 2000 and 2015. A steady increase on the number of hospitalizations occurred throughout the study period (*R* = 0.928; B = 35.649 95% CI [27.416, 43.881]), from 20 hospitalizations in 2000 to 588 in 2015 a 29.4 times increase (Table 1). Most of the hospitalizations were associated with male patients, representing 89.8% of all admissions and a total of 2,902 hospitalizations, a higher frequency when compared with all PD hospitalizations (62.7%; *n* = 42,998). The mean and median age at the time of discharge were 30.66 and 29.00 years of age, respectively, with a minimum range of 14 and maximum of 100 years. The age at discharge increased during the study period (*R* = 0.154; B = 0.399, 95% CI [0.310, 0.487]). Approximately 3.3% (*n* = 107) of the hospitalizations occurred in patients younger or with 18 years old, and the mode was 25 years with a total of 173 hospitalizations. The mean age was similar for male and female patients (males: 30.63; females: 30.69, *p* = .540). When considering all PD hospitalizations, the mean and median age were 42.00 and 44.00 years old.

**Table 1:** Hospitalizations associated with cannabis use and a primary diagnosis of psychotic disorder or schizophrenia

Year	Hospitalization episodes due to PD with associated CU (*n*)	% from all schizophrenia and other psychotic disorders hospitalizations	Hospitalizations per 100,000 inhabitants	Mean age + *SD* (years)	Male sex (%/*n*)	Mean/median LoS (days)
2000	20	0.87	0.19	24.40 + 5.64	90.0/18	19.45/9.50
2001	24	0.91	0.23	27.79 + 7.72	91.7/22	21.83/20.00
2002	41	1.53	0.39	27.29 + 5.87	97.6/40	21.71/20.00
2003	61	1.75	0.58	26.90 + 7.23	82.0/50	21.97/19.00
2004	75	2.08	0.71	28.56+ 11.15	88.0/66	29.19/21.00
2005	99	2.72	0.94	27.57 + 7.41	91.9/91	30.21/20.00
2006	82	2.23	0.78	26.99 + 8.79	90.2/74	23.67/19.50
2007	93	2.43	0.88	27.67 + 8.24	94.6/88	20.66/16.00
2008	190	3.69	1.80	30.24 + 8.06	90.5/172	15.87/11.00
2009	185	3.51	1.75	29.65 + 8.20	92.4/171	15.77/12.00
2010	211	4.11	2.00	30.86 + 9.17	89.6/189	17.03/12.00
2011	259	4.98	2.46	30.58 + 9.63	91.1/236	18.08/16.00
2012	320	5.87	3.05	31.49 + 9.64	88.1/282	19.25/14.50
2013	453	8.28	4.34	31.55 + 9.48	88.7/402	19.06/14.00
2014	532	9.60	5.13	31.59 + 9.44	89.8/478	18.08/15.00
2015	588	10.60	5.69	31.84 + 9.63	88.9/523	20.37/16.00
Total	3,233	4.71	N.A.	30.66 + 9.30	89.8/2,902	19.42/15.00

Abbreviations: CU, cannabis use; LoS, length of stay; PD, psychotic disorders.

The mean and median LoS were 19.42 and 15.00 days, respectively, and a constant decrease on the LoS was registered from 2000 to 2015 (*R* = −0.049; B = 0.308, 95% CI [−0.524, −0.092]; Table 1). The 3,233 hospitalizations represented a total estimate charge of €11.3 M during the 16 years of our study with a mean charge of €3,500 per episode.

From all the 3,233 hospitalizations, 0.5% (*n* = 16) had also an associated diagnosis code of hallucinogen abuse, 2.0% (*n* = 64) of cocaine abuse, 0.4% (*n* = 12) of amphetamine or related acting sympathomimetic abuse, and 2.0% (*n* = 65) of cocaine dependence.

When analysing all CU hospitalizations occurred in Portugal, PD or schizophrenia as primary diagnosis represented 26.4% of all episodes (*n* = 12,267 hospitalizations; Table 2). Substance‐related disorders were the second most frequent group (1,500 hospitalizations, representing 12.2%), followed by mood disorders (1,087 hospitalizations, representing 8.9%), and personality disorders (613 hospitalizations, representing 5.0%). From all the 68,630 hospitalizations with a main diagnosis of schizophrenia and other PD that occurred in Portuguese public hospitals, the percentage of hospitalizations with a CU‐related diagnosis increased from a minimum of 0.87% in 2,000 to a maximum of 10.60% in 2015, a steady and constant increase.

**Table 2:** Hospitalizations associated with cannabis use for all primary diagnosis

Year	Total hospitalizations with associated CU	Total CU hospitalizations per 100,000 inhabitants	Mean age + *SD* (years)	Male sex (%/*n*)	Mean/median LoS (days)
2000	73	0.71	27.00 + 9.86	84.9/62	12.78/6.00
2001	144	1.39	25.33 + 8.22	81.9/118	12.43/8.00
2002	210	2.01	26.73 + 7.30	69.5/146	12.85/8.00
2003	277	2.64	26.83 + 7.78	76.2/211	14.72/9.00
2004	324	3.09	28.48 + 10.24	81.5/264	16.64/10.00
2005	374	3.56	27.97 + 7.89	80.5/301	16.77/9.00
2006	347	3.29	29.38 + 9.63	82.1/285	14.94/10.00
2007	420	3.98	28.89 + 8.87	76.9/323	13.19/8.00
2008	611	5.78	30.50 + 8.79	81.3/497	13.09/9.00
2009	652	6.17	30.61 + 9.04	82.8/540	13.77/9.00
2010	823	7.78	31.84 + 10.07	80.1/659	12.21/7.00
2011	1,011	9.59	31.88 + 10.45	84.1/850	12.47/8.00
2012	1,326	12.64	32.95 + 10.88	82.4/1,093	12.89/8.00
2013	1,615	15.49	32.92 + 10.92	82.2/1,328	13.36/8.00
2014	1,944	18.74	33.68 + 11.63	83.0/1,613	12.91/9.00
2015	2,116	20.46	33.63 + 11.07	81.3/1,721	14.01/9.00
Total	12,267	N.A.	31.89 + 10.63	81.6/10,011	13.45/9.00

Abbreviations: CU, cannabis use; LoS, length of stay; PD, psychotic disorders.

## 4 DISCUSSION

PD hospitalizations linked to CU increased 29.4 times in number during the study period. This great increase was not only due to the rise on the total number of PD hospitalizations, once the percentage of episodes linked to CU also increased significantly from 2000 to 2015, reflecting the increasing weight of CU in PD hospitalizations (Table 1).

The role of cannabis in PD is still not clear, but we demonstrate an objective and increasing of CU diagnosis in PD hospitalizations. Cannabis consumption associated with dependence symptoms increased in Portugal during the study period, and its health consequences are still not clear for the consumers and the general population. The crescendo in the relative frequency of PD with an associated CU seen between 2000 and 2015 (0.87% and 10.60%) entails the increasing prevalence of cannabis consumption in patients who develop psychotic conditions, which may translate an increasing role of CU in the development of PD (Hall & Degenhardt, 2000; Ortiz‐Medina et al., 2018). At the same time, this increasing percentage of CU in PD may also correspond to the increasing detailing in administrative codification of secondary diagnosis that has been verified for other diagnostic codes in the recent years. Nevertheless, this should not be regarded strictly as a limitation once it is of great importance to understand the coding patterns of CU‐related codes (Lopez‐Pelayo, Balcells‐Olivero, & Gual‐Sole, 2013). This increase may also arise from the greater awareness of medical professionals for the possible impact of CU on the development of a PD.

The fact that the mean age of patients with PD hospitalizations associated with CU is lower than the mean age in the total group of PD hospitalizations (30.66 vs. 42.00 years old) translates the fact that a younger subgroup of the population has an associated diagnosis code of CU linked to the hospitalization episode. This demographic trend may demonstrate the possible effect that cannabis may exert by diminishing the necessary threshold to initiate a psychotic event (Miettunen et al., 2008; Roncero et al., 2018). Also, risk behaviours are clearly more associated with younger age groups, which can be a possible explanation for the different age values registered between PD hospitalizations with and without CU.

Regarding gender trends, this study shows that most of PD hospitalizations associated with CU occur in men. In Portugal, epidemiological studies already demonstrated that men had higher consume prevalence than women (14.6% vs. 4.4% for lifetime prevalence; SICAD, 2014). We now add that PD hospitalizations linked to CU occur in their vast majority in men (89.8% of all episodes). In Portugal, men are at higher risk and are more frequent users of cannabis when compared with women, a fact that may explain the results found in our study (SICAD, 2017).

In the year 2012, an epidemiologic study stated that higher risk consumers represented 7.0‰ inhabitants aged from 15 to 64 years old and that the average potency of cannabis used in Portugal increased in the last years of our study (SICAD, 2014). The increasing rate of PD hospitalizations associated with CU per 100,000 inhabitants accompanied the rate previously described in those reports. Higher consumer patterns and higher cannabis potency may be one of the reasons that could explain the increasing number of hospitalizations we found in our study (SICAD, 2017).

The mean LoS was 19.42 days, a long LoS that may refer to the need for inpatient dishabituation treatment, the treatment of organic comorbidities affected in the psychotic event, or even the possible difficulties in social reinsertion of the patient after the hospitalization. This long LoS may also explain the high mean charges associated with PD and CU hospitalizations.

### 4.1 Limitations

One of the limitations of our study is that we used an administrative database that was not previously conceived for the purpose of this project. Diagnosis codification depends on the reliability of the medical records and the quality of medical coding. In Portuguese public hospitals, the diagnosis and procedures codification is performed by trained and qualified medical doctors, which reinforces the reliability of our data. CU was evaluated by a diagnostic code and may be underrepresented due to the absence of consume information on the medical records from where coding professionals extract the data (Lopez‐Pelayo et al., 2013).

## 5 CONCLUSIONS

The use of big data is currently making its way in the field of psychiatry and mental health, and studies like ours reinforce the importance of increasing the detailing of clinical coding in administrative clinical databases. Large databases allow researchers to have a more representative sample of the studied population even though the data quality may vary depending on the health records from where the database is created (Lejoyeux et al., 2014; Simon, 2019). Although previous studies demonstrated the possible effect that cannabis may exert on brain functionality, the purpose of this study was not to prove causality but to demonstrate and describe trends regarding PD and CU. Even if one cannot establish a causal effect by analysing raw administrative data, we can conclude that the number of hospitalizations due to PD and associated with CU increased tremendously during the study period. This shift may be due to the increasing consumption of cannabis and/or the increasing codification of secondary diagnosis in administrative hospital databases. It would be interesting to know similar trends in other countries with the same or different cannabis consumption patterns.

## Appendix A. CCS number 659—Schizophrenia and other psychotic disorders 1

ICD9 CM codes:

29381 29382 29500 29501 29502 29503 29504 29505 29510 29511 29512 29513 29514 29515 29520 29521 29522 29523 29524 29525 29530 29531 29532 29533 29534 29535 29540 29541 29542 29543 29544 29545 29550 29551 29552 29553 29554 29555 29560 29561 29562 29563 29564 29565 29570 29571 29572 29573 29574 29575 29580 29581 29582 29583 29584 29585 29590 29591 29592 29593 29594 29595 2970 2971 2972 2973 2978 2979 2980 2981 2982 2983 2984 2988 2989
